# With Age Comes Representational Wisdom in Social Signals

**DOI:** 10.1016/j.cub.2014.09.075

**Published:** 2014-12-01

**Authors:** Nicola van Rijsbergen, Katarzyna Jaworska, Guillaume A. Rousselet, Philippe G. Schyns

**Affiliations:** 1 Institute of Neuroscience and Psychology, University of Glasgow, 58 Hillhead Street, Glasgow G12 8QB, UK

## Abstract

In an increasingly aging society, age has become a foundational dimension of social grouping broadly targeted by advertising and governmental policies. However, perception of old age induces mainly strong negative social biases [1, 2, 3]. To characterize their cognitive and perceptual foundations, we modeled the mental representations of faces associated with three age groups (young age, middle age, and old age), in younger and older participants. We then validated the accuracy of each mental representation of age with independent validators. Using statistical image processing, we identified the features of mental representations that predict perceived age. Here, we show that whereas younger people mentally dichotomize aging into two groups, themselves (younger) and others (older), older participants faithfully represent the features of young age, middle age, and old age, with richer representations of all considered ages. Our results demonstrate that, contrary to popular public belief, older minds depict socially relevant information more accurately than their younger counterparts.

**Video Abstract:**

## Highlights

• We model mental representations of age in young and old participants
• Young participants dichotomize age into young (like them) and old (everyone else)
• Old participants faithfully represent visual features of aging
• Inhomogeneous dark marking in the skin around the nose predicts perceived age

## Teaser

Old people have more-faithful mental representations of facial age, whereas young people have an underdeveloped mental representation that divides the age spectrum into young (like them) and old (everyone else). van Rijsbergen et al. demonstrate that the older mind depicts socially relevant information more accurately than its younger counterpart.

## Results and Discussion

The apparent age of others is widely recognized to modulate our social reactions and expectations [1, 2, 3]. The ability to accurately estimate chronological age from the face varies with one’s own age and age disparity with the observed person (the “own-age bias” [4, 5, 6]). We directly investigated the psychological basis of this effect by examining the mental representations of age in younger and older participants.

**Figure 1:**
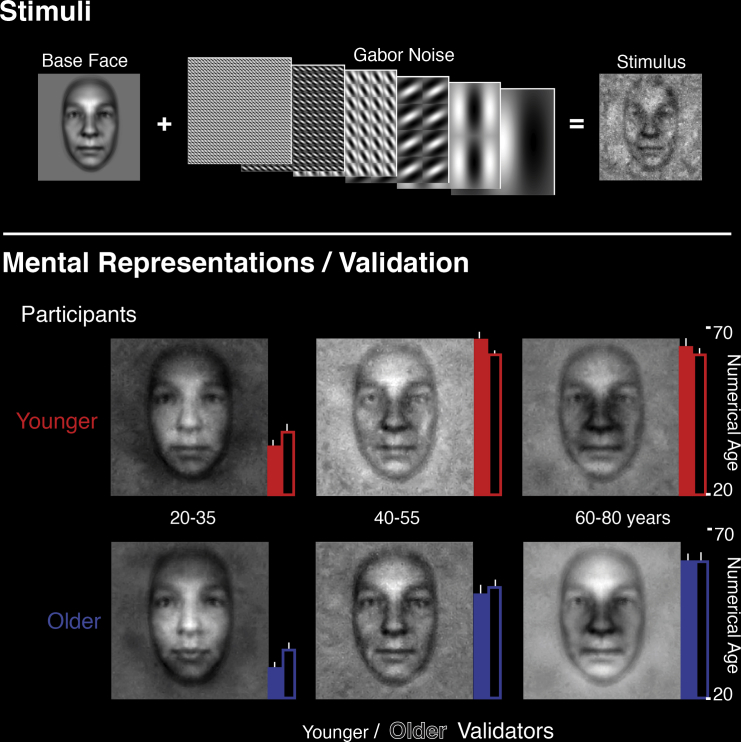
Stimulus Generation For the reverse correlation experiment, we added to a single base face random Gabor noise generated with random amplitudes of Gabor filters that recursively tiled the image across six spatial scales. Mental Representations: we averaged the mental representations across younger and older participants for the three age ranges of the reverse correlation experiment and displayed them on the same base face for display purposes. See also Figure S1 for sample individual mental representations. Validation: Younger (18–25 years) and Older (55–75 years) validators estimated the numerical age (between 20 and 70, y axis) of new base faces to which we added the mental representations from the three age ranges. Histograms report the younger (plain bars) and older (outline bars) validators’ age estimates of younger (red) and older (blue) average mental representations. Error bars indicate the SEM across participants. Tables S1 and S2 show means and SDs of the full set of data. Figure S2 shows data from individual mental representations.

We used an innovative application of reverse correlation [7, 8, 9, 10, 11] to characterize the mental representations [12, 13] of six younger (18–25 years old) and six older (56–75 years old) participants. On each experimental trial, we asked naive participants to choose one face from three simultaneously presented stimuli. Each stimulus comprised the same age-neutral base face modified by a different, randomly generated template of Gabor noise (see Figure 1, Stimuli; see Experimental Procedures). The effect of adding Gabor noise is that it perceptively changes the appearance of the age-neutral face by altering face features.

For example, consider a trial in which adding noise resulted in darkening the wrinkles extending between the nose and the mouth (see Figure 1, Stimulus). The participant might perceive this stimulus as older because darkened wrinkles correspond to their expectation of an “older face.” Thus, when the participant chooses this stimulus among the three noisy faces, we capture the information that this participant expects from an older face (e.g., another participant might expect the jowls). Over trials, we can average the chosen Gabor noise templates and add this average to the age-neutral base face to visualize the information each participant uses to estimate age.

We refer to these information images as individual “mental representations” [11, 12, 13] of age because they capture the expectations of the participant (i.e., their knowledge) of the physical appearance of an aged face—more technically, they project the participant’s knowledge of an aged face onto the parameters of a recursive organization of Gabor filters. The power of our method to study mental representations of aging is 2-fold. First, we researchers do not need to specify in an a priori manner and subsequently test the aging features that we believe participants should use to judge age, limiting researcher bias. Second, participants do not even need to be consciously aware of these aging features; as long as their age decisions systematically use face features randomly formed by the Gabor noise, the reverse correlation method will capture them, and our analyses will reveal what the features are.

We applied this approach to younger (18–25 years old) and older (56–75 years old) participants performing the choice task independently with three age ranges (20–35 years, 40–55 years, or 60–80 years). For each participant and age range, we computed an individual mental representation. We also computed six averages, one for each condition of the experimental design, to reveal the average information present in the mental representations of each age range in younger and older participants (see Experimental Procedures, Mental Representation Reconstruction). Averages emphasize the aging features common to each participant group, smoothing noise and distinctiveness due to idiosyncratic feature preferences.

To understand how younger and older participants represented age, we conducted a validation experiment that used their individual and group average mental representations as stimuli (see Experimental Procedures, Validation). We synthesized these new stimuli by adding, to novel age-neutral base faces, the mental representations (average, Figure 1; individual, Figure S1 available online) of younger and older participants, for the three age ranges. Thus, our validation stimuli were aged by the features of the mental representations of younger and older observers. We then showed these images (6 averages plus 36 individual images) to new naive participants (henceforth, validators) and asked them to numerically estimate their ages (with a number between 18 and 80; see Experimental Procedures, Validation). We found that the mental representations of older participants (blue bar in Figure 1, Validation; see also Table S1) induced numerically corresponding age estimates in all validators (11 young, 18–25 years old; 11 old, 54–79 years old), as illustrated by the monotonic increase of the validator’s age judgments (younger, plain blue; older, blue outlines) across the three age ranges—a main effect of mental representations, F(1.74, 226.8) = 1,150, p < 0.0001. In contrast, the representations of younger participants (red bars) collapsed middle age and old age into a single old category >60 years. Specifically, they induced younger (plain red) and older (red outline) validators to overestimate middle-age faces by 11 years (7.3, 11.2) (see also Figure S2 and Table S2 for the same effect with the mental representations of individual participants, and see Supplemental Information for the full repeated-measures ANOVA). We found no three-way interaction among validator age, participant age, and mental representation age range, indicating that there was no difference in discrimination ability between younger and older validators. There was, however, a small estimation bias (+3 years for younger validators).

**Figure 2:**
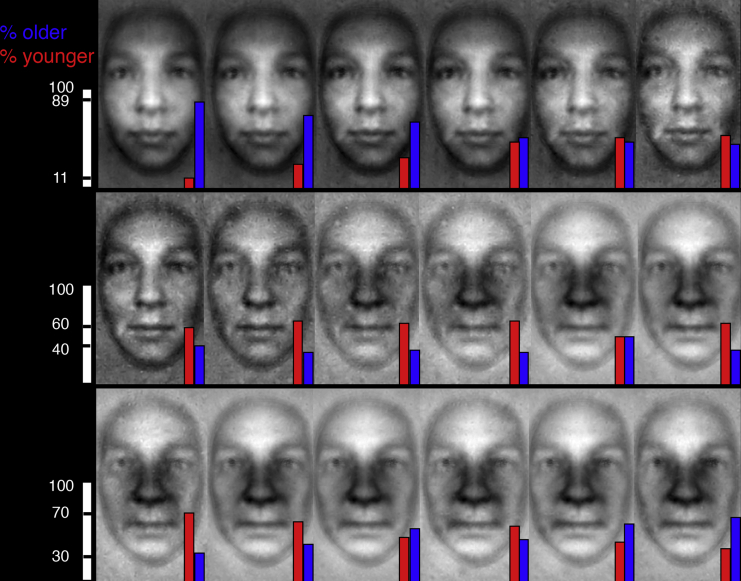
Rank Order Aging Function For each validator, we rank ordered their responses to the 36 individual mental representations in 18 bins from youngest to oldest (e.g., the first two bins contain all the representations that each validator found youngest or second youngest) and averaged them. Blue and red bars illustrate the proportion of older and younger participants’ mental representations per ranking bin. To illustrate, ranks 1 and 18 comprise mostly mental representations drawn from older participants. This implies that older participants better represent age extremes, whereas the younger participants’ representations are more compressed and central, leading to a less discriminable space.

Next, we characterized the representational space of aging as follows. For each validator, we rank ordered (in 18 ranks, from youngest to oldest) their age judgments of the 36 individual mental representations of younger and older participants that were used to construct the stimuli. Across validators, for each rank, we computed the proportion of older (Figure 2, blue bar) and younger (red bars) individual representations comprising the rank and averaged them for display (see Experimental Procedures). Figure 2 depicts the average representation corresponding to each rank, resulting in an aging function across ranks. The figure (top row) also shows that the first two ranks comprise a much greater proportion of older participants’ representations (blue bars). This indicates that older participants represent young age more faithfully, leading to the youngest numerical age judgments in younger and older validators (a similar trend applies for old age in the last two ranks). To demonstrate that the frequency distribution of younger participants’ representations diverged from that of older participants’ representations across ranks, we conducted a two-sample Kolmogorov-Smirnoff test (KS statistic [17] = 0.388, p < 0.0001; see Experimental Procedures). As the mental representations of younger participants are underrepresented in both the “youngest” and “oldest” ranks of age judgments, it necessarily follows that they concentrate around the central age ranks, thus spanning a smaller, more compressed age range, i.e., they are embedded within the representational space of the older participants. This suggests why validators could not distinguish the younger participants’ representations of the 40–55 and 60–80 age groups (cf. the color-coded histograms of Figure 1). Only a reverse correlation method can provide such direct comparative understanding of the representational spaces of age in younger and older participants.

We conclude that mental representations of aging in older participants comprise accurately interpreted age information mapping the age range, whereas younger participants’ representations are more compressed and dichotomize perceptions of age, leading to perception of two broad ranges (young, like themselves, and old).

### Specific Face Features Predict Numerical Age

**Figure 3:**
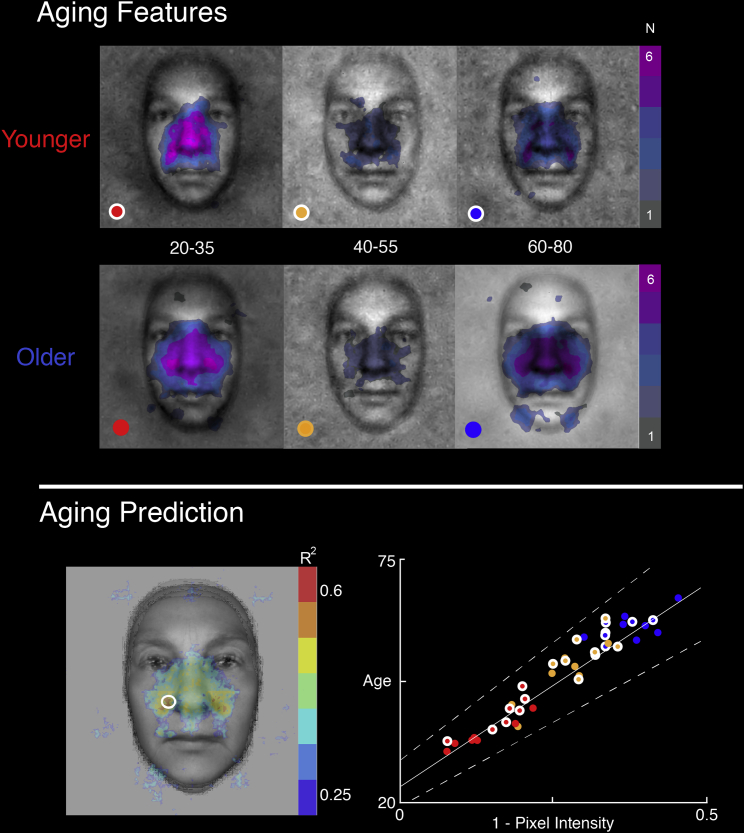
Aging Features The colored overlays highlight the significant face regions representing age in the reverse correlation experiment. The scale indicates the frequency of participants (maximum N = 6 participants in each group, Younger and Older) who mentally represented this region. Aging Prediction, left: the colors of face pixels indicate, with corresponding R^2^ values, the mental representation pixels that predict age. Maximum R^2^ values (highlighted with a white circle) identify the corners of the nose. Aging Prediction, right: at the maximum R^2^ pixel (see white circle in the face), illustration of the linear relationship between pixel intensity and age prediction, together with the maximum, minimum, and mean regression lines. Colored dots correspond to those in the top panel to indicate the age range and participant group (younger, white circle; older, plain) of some of the mental representations used as data points for age prediction. See also Figure S1 for additional illustrations of individual mental representations.

Our methods can uniquely clarify the mental representation features that predict age judgments. We computed aging features in two different ways. First, we identified the aging features common across the mental representations of individual participants [14] (see Experimental Procedures, Aging Prediction). Figure 3 (Aging Features) reveals that most participants represented older (versus younger) age with a darker (versus brighter) face center (see the 60–80 versus 20–35 panels). All participants (younger and older) also represented old age with the diagonal dark wrinkle extending from the corners of the nose to the mouth (see the 60–80 panel), whereas only older participants represented the left and right jowls in old age (see the 60–80 panel). Furthermore, there was no systematic bias for scale (i.e., spatial frequency) representations across younger and older participants, who all represented aging features mostly with the lowest two spatial frequency bands (see Figure S3). Relatedly, there was no systematic association between the upper versus lower face feature distributions across younger and older participants (see Figure S4), despite the prominent representation of the central lip areas and the jowls in older participants.

We determined which feature pixels on individual representations predict perceived age (see Experimental Procedures, Aging Prediction). Figure 3 (Aging Prediction) plots in color the pixel locations that predict aging (R^2^ > = 0.25, F(1,40) = 13, p < 0.0005), for example, those pixels darkening the corners of the nose. The white circle on the face highlights the most predictive pixel, and the rightmost panel illustrates the linear relationship between pixel intensity of the validation stimuli (color coded as in top panel) and age perception (see Figure S1 for additional data points).

Our finding that skin brightness and inhomogeneous dark marking around the nose predict perception of chronological age echoes the widespread evidence that cumulative exposure over the lifespan to solar ultraviolet light causes increase of skin pigment irregularities and decreased homogeneity of skin reflectance [15, 16]. Decreased reflectance of skin in areas of high exposure (e.g., the nose and cheeks) is correlated with chronological age, especially in UV-sensitive white Caucasian skin.

In conclusion, independent validators found that younger participants’ mental representations of age did not encompass a fully developed representational scale that enabled discrimination between middle-age and old-age groups. Comparison of the younger and older representational spaces of age revealed that the latter embedded the former, with more faithful representations of both younger and older age in older participants. We found no difference in perceptual discrimination abilities between the older and younger validators.

The dissociation between the dichotomic mental representations of aging in younger participants and the accurate perceptual discrimination of aging features in younger validators (when all information is present) warrants further investigation. At this juncture, it is worthwhile pointing out that both tasks (reverse correlation and its validation) involve perceptual judgments that are influenced by sources of information other than visual. For example, the existence of a relative social outgroup (“older people”) may elicit biases in younger participants that could differentially affect reverse correlation (when minimal information is shown) and perceptual validation (when full information is shown). A simple “own-age” effect could explain the dichotomic representations in younger participants [17]. However, older adults’ representations were richer and more accurate for both their own age groups and other age groups, ruling out the generalizability of the effect. Speculatively, we suggest that the particularly detailed older participants’ representations of young age could constitute a bias (idealization of the young), which in turn could underlie older participants’ tendency to overestimate the age of young people [2, 3, 4]. Such research questions lie at the rich intersection between available visual information and the strong biasing of categorical social perception. They deserve further investigation so that we could better understand the perceptual and social determinants of aging.

In any case, evidence of richer representations in older participants demonstrates, contrary to popular wisdom, that their minds represent socially relevant information with greater accuracy than young minds. Richer and more faithful representations of age are another example of the benefit of life experience in social cognition [18, 19, 20] and may be the product of more cross-generational experience with faces, either recent [21] or over the lifespan. Our findings warrant rigorous study of the development of mental representations across the lifespan in order to derive an objective understanding of the aging mind.

## Experimental Procedures

### Reverse Correlation

#### Ethics

This project was approved by the Glasgow University Science and Engineering Ethics Committee, reference number CSE0126.

#### Participants

Twelve participants (6 participants 18–25 years old, three females; 6 participants 56–75 years old, three females) with normal or corrected-to-normal vision participated in the experiment. Each participant gave informed written consent. We assessed older participants’ visual acuity and contrast sensitivity for normal functional range in the laboratory, on the day of the first experimental session, using a Colenbrander mixed contrast card set (see Table S3) and a Pelli-Robson chart. Participants reported no cataracts or any neurological condition and were required to have had a National Health Service eye examination within the year prior to participation. Participants over 65 were also assessed with the Montreal Cognitive Assessment (MoCa) and were all in the cognitively healthy range (>26). We recruited older participants through a local newspaper article and active-age fitness classes. We recruited younger participants through the Institute of Neuroscience and Psychology website. We compensated participants for their time at the standard rate of £6 per hour.

#### Stimuli

In each trial, we generated an experimental stimulus by adding a recursive Gabor noise mask to a base face. The base face was the average of 84 male and female face pictures (ranging from 18 to 79 years old), normalized for spatial locations of landmark features (i.e., eyes, nose, and mouth). The recursive Gabor noise mask was comprised of five cycle Morlet wavelets, at six possible orientations, in one of two polarities. We tiled the noise with these wavelets, recursively across six spatial scales, increasing the tiling density by a power of two at each spatial scale (see Figure 1, stimulus generation, which shows the systematic spatial structure of the tiling). To illustrate, the second lowest spatial frequency band is tiled with four Gabors per orientation and polarity, for a total of 48 parameters, to independently set the amplitude of each Gabor (4 Gabors × 6 orientations × 2 polarities). Consequently, in each trial, we generated three noise masks by randomly choosing the amplitude parameter of each of the 16,380 Gabor wavelets. The three noise masks were then added to the base face and simultaneously presented on the computer screen.

#### Procedure

The experiment comprised three target age ranges (20–35 years, 40–55 years, or 60–80 years), each tested with 60 blocks of 18 consecutive trials, for a total of 3,240 trials. At the start of each block, a target age range was randomly chosen from the set of possible blocks. In each of the following 18 trials, three independent noisy faces, generated as explained above, simultaneously appeared on the computer screen. We instructed participants to choose the noisy face that best fitted the target age range by pressing one of three response keys. The three faces remained on the screen until response. Participants sat in a dimly lit room, their heads maintained at 72 cm from the screen, using a chin rest. Each noisy face subtended 9.5° × 6.4° of visual angle. We ran the experiment using the Psychtoolbox-3 [22, 23, 24] for MATLAB R2012a.

#### Mental Representation Reconstruction

Reverse correlation can estimate the mental representations of the three different age ranges in younger and older participants. The logic of reverse correlation is as follows: if participants selected faces randomly across trials, then summation of the Gabor weights between −1 and 1 across trials should be near zero. In contrast, if some of the Gabor noise coincided with the participant’s mental representation of a given age range, then the participant’s choice would be biased toward the face stimuli with this Gabor noise, and the sum of Gabor weights should differ from zero. From the sum of the Gabor weights for each participant, we estimated one mental representation for each of the three age ranges of the design. Once computed, these mental representations can be reapplied to the average face (without threshold) or to new faces to visualize their aging effects. In addition, we applied a two-tailed cluster test [14] (p < 0.05, cluster size 3) to establish where the sum of the Gabor weights significantly differed from zero, using background pixels to derive the SD of the null distribution.

#### Rank Order Aging Function

For each validator (see Validation below), we rank ordered their responses to the 36 individual mental representations used to construct the validation stimuli in 18 rank bins, from youngest to oldest: the first two bins contained all the representations that each validator found youngest or second youngest. For each rank bin, we averaged its associated mental representation parameters, replotted them on the template face, and represented the proportion of representations drawn from younger (red bars) and older (blue bars) participants on each image of Figure 2. The proportions diverge mostly at the ends of the ranking scale, in the youngest and oldest age bins, which are dominated by the mental representation stimuli drawn from the older participants. The cumulative frequency distributions of young and old participants’ representation stimuli diverged across ranks, with a two-sample Kolmogorov-Smirnoff test (KS statistic = 0.38; degrees of freedom: [17]; p < 0.0001).

### Validation

#### Participants

Eleven younger validators (18–23 years old, four males) and 11 older validators (54–79 years old, five males) participated in the experiment. Recruitment and screening were identical to the reverse correlation experiment above.

#### Stimuli

We generated 12 new averaged base faces (six males) by averaging six new identities per base face; these identities differed from those averaged in the base face of the reverse correlation experiment. We generated validation stimuli either by adding to the base faces the mental representation of individual participants (3 age ranges × 6 participants × younger and older participant groups = 36 individual aging masks) or by adding the average mental representations of the group (3 age ranges × younger and older participant groups = 6 average mental representations), for a total set of 504 validation stimuli (12 base images × 42 mental representations).

#### Procedure

In each validation trial, validators saw one of the 504 validation stimuli. Validators judged the numerical age of the face by typing a two-digit number between 18 and 80. We instructed validators that the faces would span the full age range and warned them that the same facial identity might appear at different ages over the course of the experiment. At the end of the experiment, we also asked validators to judge the numerical age of the 12 average base faces, presented this time without any added mental representation, using the same procedure. Stimuli spanned 9.5° × 6.4° of visual angle and were presented one at a time on the computer screen until response, using a program written with the Psychtoolbox-3 [22, 23, 24] for MATLAB R2012a.

#### Analysis

To tease apart the aging effect of the mental representations from the aging effect of the base faces, we subtracted in each trial the perceived age of the base face from the perceived numerical age of the same base face plus mental representation. This resulted in one difference score per trial. For each validator, we took the median of these difference scores across trials, independently for each of the three age range categories. We submitted these difference scores to a repeated-measure ANOVA in SPSS (with the following factors: (1) younger versus older validators, (2) younger versus older participant mental representations, (3) mental representation age ranges, 20–35, 40–55, and 60–80, and (4) individual versus averaged mental representations). We applied the Greenhouse-Geiser correction for nonsphericity. The Supplemental Information presents the full ANOVA.

#### Aging Prediction

To determine the face features that predict the age of a face, we determined how individual face pixel intensities of the mental representations predict the validators’ age judgments. In a cross-validation, in each of 500 iterations, we randomly split the validators into two subsets. Using the first validator subset, we first computed the median age of each mental representation (average and individual representations) of the design. Then, for each face pixel, we linearly regressed the mean of the age judgments with the mean pixel intensity values of the corresponding representations. For each face pixel, this parameterized a linear model. To cross-validate the model, we used the second subset of validator judgments and computed for each pixel the R^2^ measure of fit between the linear model and the new data (for a total of 500 R^2^ measures of fit per pixel). Figure 3 (Aging Prediction, left panel) shows the minimum predictive R^2^ value (computed over the 500 measures) of each pixel (R^2^ > = 0.25, F(1,40) = 13, p < 0.0005). The white circle at the nose wrinkle shows the pixel with the highest predictive power. For this pixel, the right panel illustrates the linear fit between pixel intensities (x axis) and mean age judgment (y axis). Dotted lines represent the range of the regression parameters over the 500 iterations.

## Supplemental Information

Document S1. Supplemental Experimental Procedures, Figures S1–S4, and Tables S1–S3

Document S2. Article plus Supplemental Information
