# Supplementary material for: With Age Comes Representational Wisdom in Social Signals
Source: Curr Biol. 2014 Dec 1;24(23):2792–6. doi: 10.1016/j.cub.2014.09.075 (PMC4251953; doi:10.1016/j.cub.2014.09.075)
Supplement: Document S1. Supplemental Experimental Procedures, Figures S1–S4, and Tables S1–S3 [file mmc1.pdf]

Current Biology, Volume *24*

Supplemental Information

With Age Comes Representational Wisdom in Social Signals

Nicola van Rijsbergen, Katarzyna Jaworska, Guillaume A. Rousselet, and Philippe G. Schyns

Supplemental Materials:

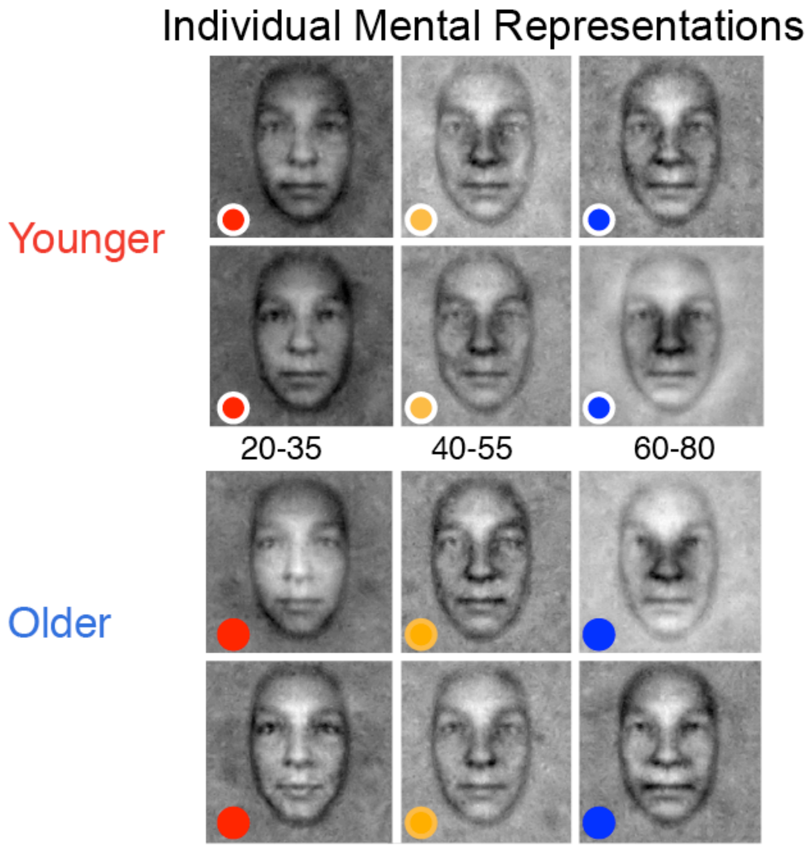

Figure S1, related to Figure 1 and Figure 3. Examples of the mental representations of four individual participants (2 young, 2 old) for the three age ranges tested in the reverse correlation experiment. The colored dots on each mental representation correspond, for mental representations of individual participants, to some of the data points plotted on and around the regression line of Figure 3, Aging Prediction.

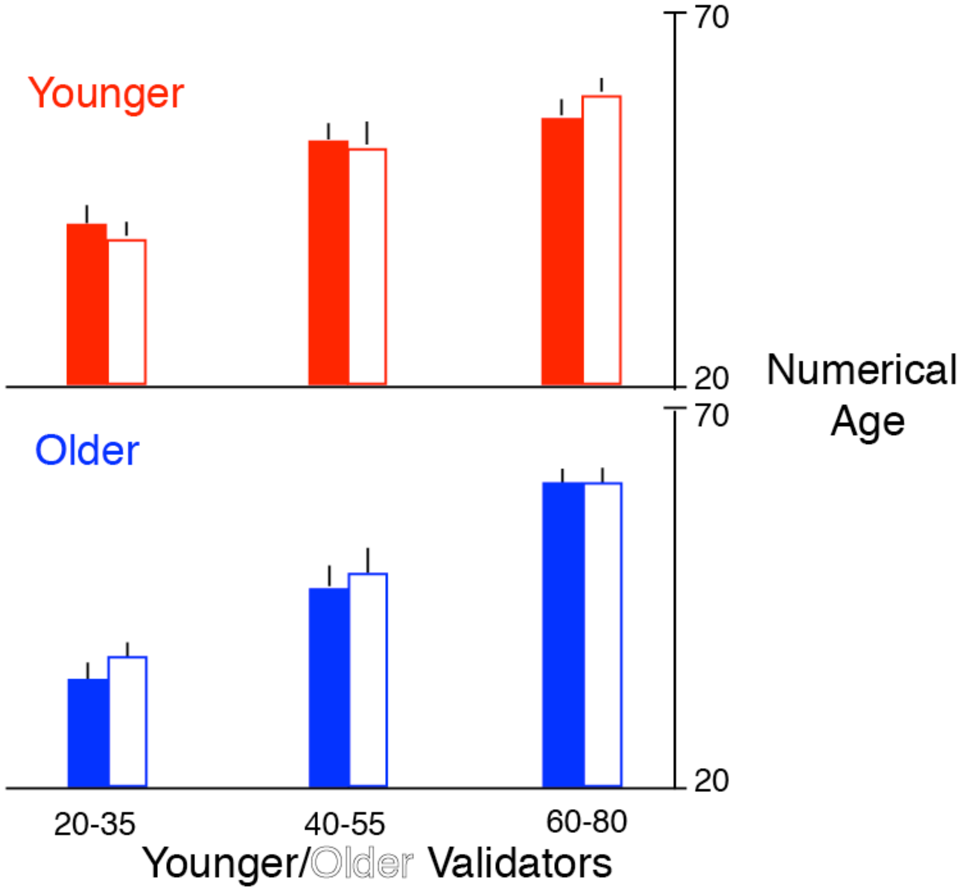

Figure S2; related to Figure 1: Validation of the individual mental representations derived from the reverse correlation experiment. Younger (18-25 years) and older (55-75 years) validators were instructed to judge the numerical age (between 20 and 80, y-axis) of new base faces to which we added the individual mental representations from the three age ranges (x-axis) derived in the reverse correlation experiment. The histograms indicate the age judgments of younger (plain bars) and older (outline bars) validators when shown faces aged with the individual mental representations of younger (red) and older (blue) participants. Error bars indicate the standard error of the mean across participants.

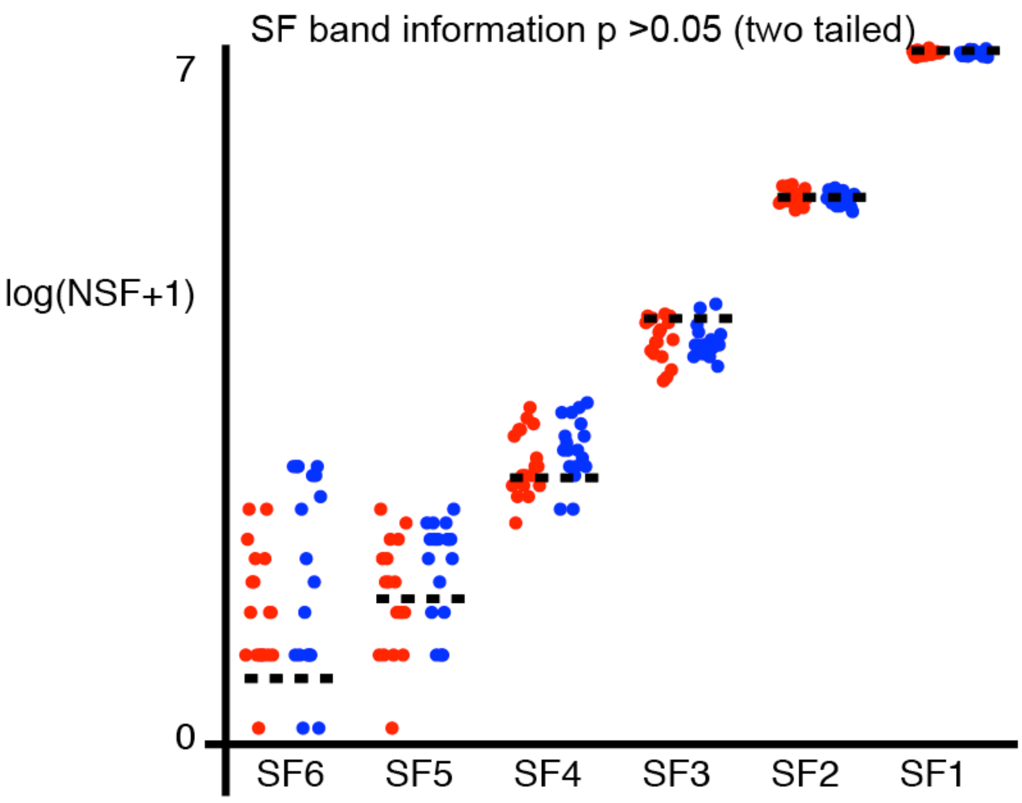

Figure S3, related to Figure 3: Spatial Frequency Composition of Mental Representations.
In this analysis we tested the possibility that older observers might rely on a different range of spatial frequency information to solve the categorization task. Specifically, we were interested in the possibility that older observers might show a bias towards lower special frequency information.

For each participant and mental representation, we calculated a percentile threshold ($p<0.05$, two-tailed) from the total distribution of amplitude parameters of the noise Gabors across all 6 Spatial Frequencies (SF) bands. If older and younger participants both used information at all SF, we would expect a linear increase in the log of the number of above threshold parameters (represented here with the 5% threshold dotted black line) together with increasing SF band. SF6 is lowest spatial frequency, that has only one Gabor per orientation and phase, and SF1 is the highest spatial frequency with 1024 Gabors per orientation and phase. The red (younger participants) and blue dots (older participants) represent the log of the actual number of SF (NSF) parameters above threshold at each scale. Both older and younger participants show proportionally more representation of lower SF information (i.e. SF6 to 4) than higher SF. Critically, there is no difference between younger and older participants at any SF level--Wilcoxon test for difference in medians at lowest SF level = 373, $z = 1.27$, $p = 0.2$.

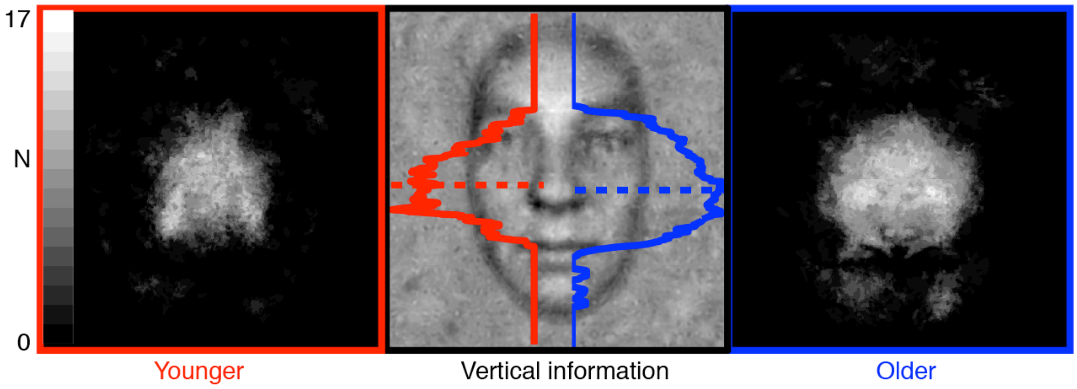

Figure S4, related to Figure 3: Spatial Distributions of Visual Information in the Mental Representations.
In this analysis we considered the possibility that older observers might show a spatial bias in the locations of features they selected to solve the categorization task: specifically whether they might have showed a bias to the upper or lower face.
The red box shows the spatial distribution of above threshold pixels (cluster test, p = 0.05, two-tailed) concatenated across all individual younger participants' representations of all age ranges. Plotted against face features, the red line on the central gray scale image shows the profile of the distribution on the vertical axis of threshold pixels with the center of the distribution shown as a dashed line. The blue box and lines illustrate the same information for the older participants. The distributions are centered five pixels apart (270 and 265 on the Y axis). Older participants show more information usage of the center lip area, and of the jowl area, whereas younger participants use the nose wrinkle area that extends to the lip area.

Supplemental Data related to Figure 1 and Figure 3.

Table S1, related to Figure 1 and Figure 3, shows the mean of median age judgments +/- standard deviations for the younger and older validators for (i) older participants' mental representations, and (ii) younger participants' mental representations.

S1(i) Older participants' mental representations

Validators	Level	20-35	40-55	60-80
Older	Averaged	28 +/- 6	48 +/- 10	57 +/- 7
	Individual	37 +/- 8	48 +/- 9	60 +/- 6
Younger	Averaged	33 +/- 7	50 +/- 7	57 +/- 8
	Individual	34 +/- 9	46 +/- 11	60 +/- 7

S1(ii) Younger participants' mental representations

Validators	Level	20-35	40-55	60-80
Older	Averaged	33 +/- 6	62 +/- 6	60 +/- 8
	Individual	39 +/- 10	51 +/- 10	58 +/- 6
Younger	Averaged	37 +/- 8	58 +/- 4	58 +/- 5
	Individual	41 +/- 10	52 +/- 7	55 +/- 7

Table S2, related to Figure 1 and Figure 3, shows the mean of median age judgments expressed as difference scores, +/- standard deviations, where the validator age-ratings of the base faces have been subtracted on each trial from the perceived age of the base face plus mental representation, for older(i), and younger(ii) participants' mental representations.

S2(i) Base faces subtracted: older participants' mental representations

Validators	Level	20-35	40-55	60-80
Older	Averaged	-22 +/- 10	-2 +/- 10	4 +/- 4
	Individual	-18 +/- 11	-5 +/- 11	6 +/- 7
Younger	Averaged	-17 +/- 6	0 +/- 7	8 +/- 8
	Individual	-12 +/- 8	-1 +/- 9	9 +/- 9

S2(ii) Base faces subtracted: younger participant mental representations

Validators	Level	20-35	40-55	60-80
Older	Averaged	-16 +/- 9	8 +/- 7	7 +/- 5
	Individual	-12 +/- 10	0 +/- 3	4 +/- 8
Younger	Averaged	-13 +/- 8	7 +/- 7	10 +/- 7
	Individual	-7 +/- 8	2 +/- 7	5 +/- 7

Supplemental Experimental Procedures

Validation: Full ANOVA:

We performed a repeated measure ANOVA in SPSS, with a between-subject factor, validator age, and three within-subject factors: participant age (younger vs. older), mental representation's age range (20-35, 40-55, 60-80), representational level (individual vs. averaged mental representations).

Younger and older validators brought small but significantly different biases to the task. Compared to younger validators, older validators estimated face age 3.2 years younger (SEM 1.06), $F(1,130) = 8.96$, $p < 0.0001$. Mental representations of younger participants were on average judged older (by 3.9 mean years, 95% confidence interval [3.4, 4.4]) than mental representations of older participants, $F(1,130) = 236$, $p < 0.0001$. There was a main effect of increasing age judgment with age range, $F(1.74,226.8) = 1150$, $p < 0.0001$. The 20-35 mental representations made the base faces look younger (younger participants, -12 [-13.7, -10.8] years; older participants, -17 [-18.5, -15.7] years), whereas the 60-80 mental representations made the base faces look older (younger participants 6.6 [5.7, 8] years; older participants 6.8 [5.7, 7.5] years). However, the middle-age 40-55 mental representations made the faces look older if they were from younger participants, +4.6 [3.5, 5.8] years, but younger if they were from older participants -2 [-3.4, -0.5] years.

The interaction between the age range of the mental representations and the age of participants was significant, $F(1.84, 240.1) = 42$, $p < 0.0001$. Three Bonferroni t-tests between mental representations from the older and younger participants in each age range demonstrated the nature of the interaction. The 40-55 mental representations of younger participants were significantly different from those of older participants by 5.1 [3.9, 6.2] years, paired t-test $t(21) = 9.1$, $p < 0.05$. Similarly the 20-35 mental representations from younger participants led to significantly ($p < 0.05$) older judgments of 4.5 [3.4, 5.7] years compared with those of older participants, paired t-test, $t(21) = 8.1$. However, the effect was reversed for the 60-80 age range masks, -2.3 [-0.8, -3.9] years, $t(21) = -3.1$.

There was no main effect of individual vs. averaged mental representations. However, there was an interaction between individual vs. averaged, and mental representations range, $F(1.91, 251)= 171$, $p < 0.0001$, suggesting enhanced aging effects in the average mental representations. Average mental representations from the 20-35 range were judged to be younger (-5 [-3.6, -6] years) than individual mental representations, while average mental representations from the 40-55 range were judged older by 5 [3.5, 7.1] years. The 60-80 average mental representations were judged only slightly older (0.3 years) than the individual representations.

Table S3. Older Observer Screening criteria and age range (applicable to Experiments 1 and 2)

Test	Cutoff criteria	Expt. 1<br>Median, Range	Expt, 2<br>Median, Range
Montreal Cognitive Assessment 64>years	26/30 (normal cognitive function)	29 [26, 30]	28 [26, 30]
High Contrast card 63cm	<90 (normal to very mild impairment)	101 [96, 104]	96 [90,105]
Low Contrast 63 cm	< 83 (normal to very mild impairment)	90 [87, 98]	89 [83, 95]
Contrast sensitivity	1.95 (normal)	1.95	1.95
Age	>54 years	67 y.o. [56, 75]	68 y.o. [54, 79]
